# Management of Pseudomonas Urinary Tract Infections (UTIs) in the Context of Extensive Antibiotic Anaphylaxis: A Case-Based Perspective

**DOI:** 10.7759/cureus.85461

**Published:** 2025-06-06

**Authors:** Ryan N Gibson, Ben Hur Aguilar

**Affiliations:** 1 College of Medicine, Drexel University College of Medicine, Philadelphia, USA; 2 Department of Internal Medicine, Bayhealth Medical Center, Milford, USA

**Keywords:** allergy and anaphylaxis, antibiotics susceptibility, antimicrobial resistance, pseudomonas aeuginosa, uti

## Abstract

This case report discusses a 56-year-old African American woman with a history of recurrent urinary tract infections (UTIs) and repeated anaphylactic reactions to multiple antibiotics. She presented with dysuria and foul-smelling urine but denied fever or catheter use. Notably, she had a recent hospitalization for methicillin-resistant *Staphylococcus aureus* (MRSA) skin infection of the scalp. Physical examination revealed right costovertebral angle (CVA) tenderness. Urine dipstick showed positive nitrites and large leukocyte esterase. Culture identified *Pseudomonas aeruginosa *(*P. aeruginosa*), sensitive to several intravenous antibiotics, including aztreonam. Due to her extensive antibiotic allergies, she was referred to the emergency department, where she received IV aztreonam with resolution of her symptoms. This case highlights the clinical challenge of managing *Pseudomonas* UTIs in patients with limited antibiotic options and underscores aztreonam as a valuable therapeutic alternative in such settings. It also emphasizes the importance of individualized antibiotic oversight in patients with complex allergies.

## Introduction

Urinary tract infections (UTIs) are among the most common infections encountered in both outpatient and inpatient settings. Epidemiological data indicate that 50% to 60% of women will experience at least one UTI during their lifetime [1,2]. While community-acquired UTIs are common, catheter-associated urinary tract infections (CAUTIs) represent a significant proportion of nosocomial infections, accounting for approximately 40% of hospital-acquired infections [2]. The pathophysiology of CAUTIs involves disruption of the natural flushing mechanism of urine, which facilitates bacterial adherence and colonization [2].

Although *Escherichia coli *(*E. coli*)* *accounts for approximately 75% of community-acquired UTIs, other pathogens, such as *Pseudomonas aeruginosa *(*P. aeruginosa*), though less common, pose unique diagnostic and therapeutic challenges. *Pseudomonas*, responsible for approximately 1% of community-acquired UTIs, is notable for its resistance patterns and limited antibiotic susceptibility [1,2].

We present a case of a 56-year-old female patient with recurrent UTIs and severe antibiotic allergies who developed a *Pseudomonas *UTI and was successfully treated with aztreonam in the inpatient hospital setting.

## Case presentation

A 56-year-old African American female patient with a history of recurrent UTIs presented to the clinic with right lower back pain, urinary frequency, dysuria, and foul-smelling urine that started three days prior to presentation. She denied fever, chills, or flank pain. She noted that she had been hospitalized a month prior for methicillin-resistant *Staphylococcus aureus* (MRSA) infection of the scalp, treated with linezolid, and denied having a urinary catheter placed at that time. She was not immunocompromised. She had three UTIs within the past year. Each episode had consistently cultured *E. coli* and was previously treated with fosfomycin.

The patient reported an extensive history of antibiotic allergies, including anaphylactic reactions to penicillins, fluoroquinolones, sulfonamides, tetracyclines, cefdinir, and nitrofurantoin. On physical examination, she was afebrile with stable vital signs and in mild distress due to pain. The right costovertebral angle (CVA) tenderness was noted. Urinalysis showed positive nitrites and a large amount of leukocyte esterase. Given the history and physical exam findings, there was concern for pyelonephritis. Urine and blood cultures were sent, and a renal ultrasound was completed, which revealed no evidence of infection, hydronephrosis, or renal calculi.

Given her history of recurrent *E. coli *UTIs, a preliminary diagnosis of *E. coli *UTI was made, and she was prescribed fosfomycin empirically while awaiting culture results.

Two days later, blood cultures were negative; however, urine culture grew 10,000-50,000 colony-forming units (CFU) per mL of *P. aeruginosa*, susceptible to amikacin, aztreonam, cefepime, ciprofloxacin, meropenem, and piperacillin-tazobactam (Zosyn). Results are summarized in Table 1.

**Table 1:** Susceptibility of Pseudomonas aerguinosa MIC: minimum inhibitory concentration MIC is the lowest concentration of an antibiotic that prevents visible growth of a microorganism. This helps to determine a pathogen's susceptibility to a particular antibiotic.

Antibiotic	*Pseudomonas aerguinosa* (MIC)
Amikacin	≤8 mg/L: Susceptible
Aztreonam	=8 mg/L: Susceptible
Cefepime	=4 mg/L: Susceptible
Meropenem	≤0.5 mg/L: Susceptible
Piperacillin + Tazobactam	8/4 mg/L: Susceptible

The patient was contacted with the results and reported she had been unable to fill the fosfomycin prescription due to insurance denial. Given her extensive antibiotic allergies, she was advised to present to the emergency department for further management. Upon presentation to the emergency department, she completed a CT scan of the abdomen and pelvis, which showed no signs of pyelonephritis. The patient was ultimately admitted to the hospital for intravenous antibiotics. 

In the hospital, she was co-managed by the Infectious Disease (ID) service. After consideration of the organism's susceptibility, the patient's allergy history, and potential side effect risks, the patient was treated with a five-day course of intravenous aztreonam (2 g every eight hours). She tolerated the medication well, with no allergic reaction, and experienced complete resolution of her urinary symptoms by the end of the treatment course.

## Discussion

**Figure 1:**
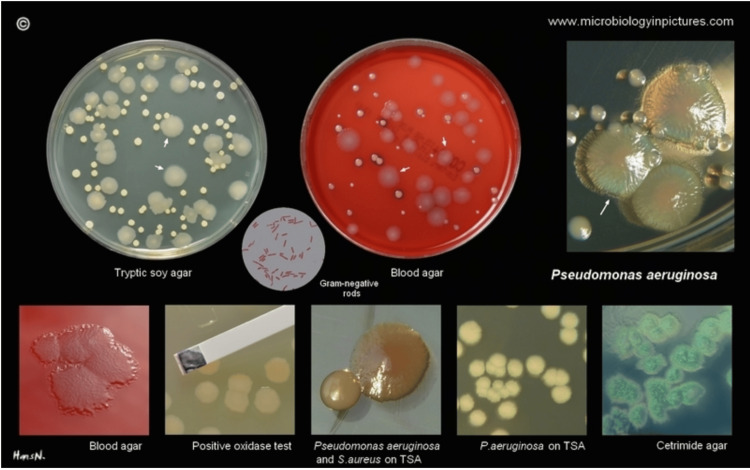
Pseudomonas plated Reproduced with permission from Hans Newman, www.microbiologyinpictures.com. Permission for the use of this image has been granted by the publisher [3].

UTIs are one of the most common infections of the body [1,2]. Hallmark symptoms consist of dysuria, or painful urination, and malodorous urine. In patients showing signs of a UTI, obtaining a urine culture is a key diagnostic step. Culturing, typically performed using agar plating techniques, enables identification of the causative organism and determination of its antimicrobial susceptibility profile. This step is particularly important in individuals with risk factors for multidrug-resistant or atypical pathogens, such as *P. aeruginosa*. Shown in Figure 1 is an example of *P. aeruginosa* growth on agar, illustrating its characteristic colony morphology [3].

Despite UTIs being common, *P. aeruginosa* remains a relatively uncommon causative agent [4-6]. Risk factors for *P. aeruginosa* UTIs include structural abnormalities of the urinary tract, recent antibiotic use, prior hospitalization, and the presence of urinary catheters [4,7]. Treatment of *Pseudomonas *infections typically involves aminoglycosides, fluoroquinolones, beta (β)-lactams, or monobactams [5]. However, due to the organism’s intrinsic resistance mechanisms and increasing adaptability, it is becoming more difficult to treat with these standard agents [4]. A key contributor to this resistance is *Pseudomonas*’ ability to form biofilms, which protect bacterial colonies from antibiotics and the host immune response, making eradication even more challenging [8]. Table 2 demonstrates a summary of potential ways of resistance [8].

**Table 2:** A summary of how antimicrobial resistance may develop Source: [8]

Adaptive resistance	Intrinsic resistance	Acquired resistance
Biofilm formation	Decreased outer membrane permeability	Porin mutations
	Antibiotic inactivating enzymes	Horizontal gene transfer
	Efflux pump	Mutations in the antibiotic target

Given these challenges, selecting an appropriate antibiotic and treatment duration is critical. Delayed or inadequate treatment may lead to complications such as pyelonephritis, urosepsis, and increased antimicrobial resistance [7]. In this case, the patient had experienced three episodes of UTIs within the past year and had a recent hospitalization requiring antibiotics, factors likely contributing to the emergence of a *Pseudomonas *infection, as this most likely caused a disruption of the patient's normal flora and created a selective environment that favored the growth of *Pseudomonas*. Although the urine culture revealed broad antibiotic susceptibility, oral treatment options were limited due to the patient’s history of anaphylaxis to several antibiotic classes. Based on the organism’s susceptibility profile, the patient’s allergy history, and potential side effect risks, intravenous aztreonam was selected. Aztreonam is currently the only FDA-approved monobactam antibiotic. It functions by inhibiting bacterial cell wall synthesis through binding to penicillin-binding protein 3 (PBP-3) [9,10]. It is active primarily against aerobic Gram-negative bacteria, including many Enterobacteriaceae, *Haemophilus influenzae*, and *Neisseria *species, including strains that produce β-lactamase enzymes [9,11]. While it lacks efficacy against Gram-positive organisms and anaerobes, aztreonam is generally well-tolerated and is considered safe in patients with penicillin or cephalosporin allergies due to its low immunogenicity [11]. Unlike aminoglycosides, aztreonam does not carry risks of nephrotoxicity or ototoxicity, and it has not been associated with coagulopathies [11]. However, monitoring of liver function is recommended due to the potential for hepatotoxicity. Other adverse effects include nausea, vomiting, rash, and phlebitis at the injection site [11]. In this case, a five-day course of 2 g IV aztreonam every eight hours resulted in complete resolution of symptoms without adverse reactions. This case underscores the importance of personalized antibiotic selection in managing *Pseudomonas *UTIs, particularly in patients with extensive drug allergies. It also highlights aztreonam as a valuable therapeutic option in such challenging scenarios.

## Conclusions

This case highlights the complexities involved in diagnosing and managing a *Pseudomonas *UTI in a patient with anaphylaxis to multiple antibiotics. *Pseudomonas *poses significant treatment challenges due to its intrinsic resistance mechanisms and limited susceptibility to standard therapies. These challenges are compounded in patients with constrained antibiotic options due to anaphylactic reactions. In this case, recent hospitalization and prior antibiotic use likely contributed to a disruption of the patient’s normal flora, creating a selective environment that favored *Pseudomonas *over more typical pathogens like *E. coli*. Despite broad susceptibility, treatment options were severely limited due to the patient's extensive allergy history. Aztreonam, a monobactam with minimal cross-reactivity in beta-lactam-allergic individuals, was selected and led to complete clinical resolution without adverse effects. This case underscores aztreonam as a valuable therapeutic option in similarly challenging scenarios. It also reinforces the need for an individualized, patient-centered antibiotic approach, particularly in patients with recurrent infections and complex allergy profiles. Early ID consultation, appropriate diagnostic workup, and consideration of a referral to an allergist for desensitization may help broaden future treatment options and improve outcomes.
